# Bispecific Antibodies in Hematologic Malignancies in the Outpatient and Community Settings

**DOI:** 10.1155/ah/4529571

**Published:** 2026-05-07

**Authors:** Amrit S. Gonugunta, Asad Haider, Virginia Mohlere, Mahmoud Aljurf, Shahrukh Hashmi, Muhammad Bilal Abid

**Affiliations:** ^1^ Department of Internal Medicine, The University of Texas Health Science Center, Houston, Texas, USA, uth.edu; ^2^ Division of Hematology/Oncology, The University of Texas Health Science Center, Houston, Texas, USA, uth.edu; ^3^ Oncology Center, King Faisal Specialist Hospital Center & Research, Riyadh, Saudi Arabia, co.bydgoszcz.pl; ^4^ Department of Medicine, Sheikh Shakhbout Medical City, Abu Dhabi, UAE, ssmcabudhabi.ae; ^5^ Mayo Clinic Cancer Center, Mayo Clinic, Rochester, Minnesota, USA, mayo.edu; ^6^ College of Medicine and Health Sciences, Khalifa University, Abu Dhabi, UAE, kustar.ac.ae; ^7^ Cellular Immunotherapy and Blood & Marrow Transplant Program, Hematologic Malignancy Section, School of Medicine, Texas Tech University Health Science Center, Lubbock, Texas, USA

**Keywords:** BiTEs, BsAbs, CAR-T therapy, community practice, CRS, ICANS, infections, outpatient, toxicities

## Abstract

Bispecific antibodies (BsAbs) are novel antitumor agents that simultaneously engage tumor‐associated antigens with CD3 on endogenous effector T cells, leading to cancer cell death. The immune‐mediated toxicity rates associated with BsAbs are considerably lower than those seen with CAR T‐cell therapy. Hence, widespread utilization of BsAb products in outpatient and community settings will allow greater and more homogeneous access and thus improve patient outcomes. In this review, we discuss the unique toxicities associated with BsAbs currently approved by the U.S. Food and Drug Administration and provide a disease‐agnostic framework for the safe community administration of BsAb therapy. With academic, community, and industrial partnerships, broader deployment of BsAbs could be streamlined. Education, provision of in‐services, leveraging protocols and experiences, and development of templates and guidelines will make this transition smoother and allow every patient in need of BsAbs to receive timely access to these survival‐prolonging therapies.

## 1. Introduction

Bispecific antibodies (BsAbs) are novel antitumor therapies that engage tumor‐associated antigens with CD3 on endogenous effector T cells [1]. This concurrent binding facilitates direct T‐cell activation and tumor destruction [2]. Data from landmark clinical trials have resulted in the approval of BsAbs for several cancers, with further efforts underway to expand indications to other tumor types [3–9]. Currently, a large number of BsAbs exist in the clinical pipeline, and a majority are being evaluated in patients with cancer [10]. Research into these medications has led to their approval by the U.S. Food and Drug Administration. As of 2023, 14 of these medications have been approved, with 11 utilized for the treatment of refractory and progressive cancers [1].

While novel BsAb therapy has significantly improved outcomes, concerns exist regarding the financial and logistical constraints associated with these medications. These trends may influence decision‐making, delay care, and negatively impact the psychosocial well‐being of patients, which may have a downstream impact on treatment outcomes [11, 12]. Furthermore, these trends may have disproportionate impacts on vulnerable populations, including those of lower socioeconomic status, those of younger age at cancer diagnosis, and those who are under‐ or uninsured [13].

Currently, data on the financial toxicity of BsAbs are limited. However, extrapolation of expense‐related literature from other engineered T‐cell products suggests that BsAbs will have the potential to cause financial strain for many patients, given the costs and indefinite duration of BsAb therapy [14–16].

Previous studies have demonstrated that outpatient administration of several other types of cancer therapy is associated with better healthcare utilization, reduced financial expenditures, and improved quality of life compared with inpatient administration [17, 18]. Given these findings for other cancer treatments, similar changes can be expected for outpatient utilization of BsAbs. Previous studies have demonstrated that other T‐cell–engaging therapies are feasible in the outpatient setting, particularly with chimeric antigen receptor (CAR) T‐cell therapy [19, 20]. These previous studies pave the way for BsAbs in the outpatient and community settings.

Transitioning BsAb therapy from inpatient to outpatient therapy is not a novel idea, as other antineoplastic agents have demonstrated feasibility in the outpatient setting [19–21]. Herein, we propose an outpatient model for BsAb utilization and discuss the nuances associated with the outpatient administration of BsAbs, including barriers to initiation and administration of BsAbs in the outpatient setting. Finally, we examine the toxicities associated with these medications and propose strategies to mitigate the toxicities to optimize the widespread utilization of BsAbs in community settings.

## 2. Toxicities and Risk Management

Clinical trials and real‐world data have demonstrated early immune‐mediated toxicities, delayed immune reconstitution, and infectious complications associated with BsAbs. Pivotal trials demonstrated grade ≥ 3 cytokine release syndrome (CRS) rates between 0% and 4% and grade ≥ 3 immune effector cell–associated neurotoxicity syndrome (ICANS) rates between 0% and 3% [22]. These complication rates are considerably lower than those seen with the CAR T‐cell therapy, setting the premise for safer and widespread utilization of BsAb products in the outpatient and community settings (Tables 1 and 2). Consensus recommendations report that rates of tumor lysis syndrome (TLS) are not significantly different from other therapies [23]. TLS is an adverse event infrequently reported in landmark trials for BsAbs. Consensus guidelines recommend consideration of clinical characteristics such as disease volume, histology, and renal function.

**TABLE 1:** Trial data of bispecific antibody therapy for multiple myeloma.

Drug	Teclistamab	Elranatamab	Linvoseltamab^a^	Talquetamab^c^
Trial	MajesTEC‐1	MagnetisMM‐3	LINKER‐MM1	MonumenTAL‐1
Author	Moreau 2022	Lesokhin 2023	Bumma 2024	Chari 2023
Route of administration	SQ	SQ	IV	SQ
Dose, dosing schedule	1.5 mg/kg weekly after step‐up dosing, with persistent responders switching to every other week dosing	76 mg weekly after step‐up dosing, with persistent responders switching to every other week dosing	50 mg weekly after step‐up dosing 200 mg weekly after step‐up dosing	405 μg (weekly, every other week, or monthly) 800 μg (weekly, every other week, or monthly)
Target antigen	BMCA	BMCA	BCMA	GPRC5D
ORR	63%	61%	48%, 71%	70%, 64%, 72%
CR	39%	35%	21%, 50%	23%, 23%, 17%
mPFS	11.3	NR	NR, NR, NR	NR, NR, NR
CRS	119 (72.1%)	71 (57.7%)	57 (55%), 54 (46%)	23 (77%), 35 (79.5%), 50 (49%)
Grade ≥ 3 CRS	1 (0.6%)	0 (0%)	2 (2%), 1 (1%)	1 (3%), 0 (0%), 5 (4.9%)
Onset, median (range)	2 (1–6) days	2 (1–9) days	4.4 (−0.6–40.8) hours^b^ 11 (−1.1–183.6) hours^b^	2 (1–22) days 2 (1–5) days 1 (1–3) days
Duration, median (range)	2 (1–9) days	2 (1–19) days	13 (1.0–104.1) hours 15.6 (1.0–96.0) hours	2 (1–3) days 2 (1–5) days 2 (1–9) days
Treatment	Tocilizumab: 60 (36.4%) Steroids: 14 (8.5%)	Tocilizumab or Siltuximab: 27 (22.7%) Steroids: 10 (8.4%)	Tocilizumab: 26 (25%) Steroids: 15 (14%) Tocilizumab: 22 (19%) Steroids: 13 (11%)	Tocilizumab: 20 (66.6%), Steroids: 1 (3.3%) Tocilizumab: 32 (58.1%), Steroids: 5 (9.1%) Tocilizumab: 38 (37.3%), Steroids: 11 (10.8%)
ICANS	24 (14.5%)	4 (3.4%)	NR, 9 (8%)	3 (10%)^d^, 2 (5%), 6 (5.9%)
Grade ≥ 3	1 (0.6%)	0	NR, 3 (3%)	0, 0, 3 (2.9%)
Neutropenia	117 (70.9%)	60 (48.8%)	30 (29%), 50 (43%)	20 (67%), 16 (36.4%), 48 (47.1%)
Grade ≥ 3	106 (64.2%)	60 (48.8%)	28 (27%), 49 (42%)	18 (60%), 14 (31.8%), 27 (26.5%)
Infections	126 (76.4%)	86 (69.9%)	NR, 87 (74%)	14 (47%), 15 (34%), NR
Grade ≥ 3	74 (44.8%)	49 (39.8%)	NR, 42 (36%)	2 (7%), 3 (7%), NR

*Note:* These agents were each approved in relapsed/refractory multiple myeloma for patients who have already received ≥ 4 lines of therapy, including protease inhibitors, immunomodulatory agents, and CD38 inhibitors. Data are no. (%) unless otherwise indicated. ICANS, immune effector cell–associated neurotoxicity syndrome; IQR, interquartile range; IV, intravenous; SQ, subcutaneously.

Abbreviations: CR, complete response; CRS, cytokine release syndrome; FDA, Food and Drug Administration; mDOR, median duration of response; mPFS, median progression‐free survival; NR, not reported; ORR, overall response rate; RR, relapsed/refractory.

^a^For the linvoseltamab trials, there were 2 dosing protocols; responses, adverse events, and treatments are reported from left to right for the 50 and 200 mg groups.

^b^Negative numbers indicate CRS onset prior to the end of linvoseltamab administration.

^c^For the talquetamab trials, there were 3 dosing protocols; responses, adverse events, and treatments are reported in descending order or from left to right for the 405, 800 μg, and IV groups.

^d^Reported as neurotoxicity.

**TABLE 2:** Trial data of bispecific antibody therapy for non‐Hodgkin lymphoma.

Drug	Glofitamab	Epcoritamab	Mosunetuzumab	Odronextamab
Author	Dickinson 2022	Thieblemont 2023	Budde 2022	Bannerji 2022
Route of administration	IV	SQ	IV	IV
Duration	Time‐limited (12 cycles)	Until PD/AE	Time‐limited (CR: 8 cycles; PR: 17 cycles)	TBD
Dose; dosing schedule	30 mg IV after step‐up dosing and pretreatment with obinutuzumab 1000 mg	48 mg SQ after step‐up dosing	30 mg IV after step‐up dosing	Step‐up dosing, with target dosing ranging 0.1–320 mg IV
Target antigen	CD20	CD20	CD20	CD20
ORR	52%	63%	80%	NR
CR	39%	39%	60%	NR
mPFS	3.9	4.4	17.9	NR
mDOR, months	18.4 NR for CR	12 NR for CR	22.8 NR for CR	NR
CRS	97 (63%)	78 (49.7%)	40 (44%)	89 (61%)
Grade ≥ 3	6 (4%)	4 (2.5%)	2 (2%)	10 (7%)
Onset, median (range)	13.6 (6.2–51.8) hours	NR	5 (IQR, 3–9) hours	NR
Duration, median (range)	30.6 (0.5–316.7) hours	NR	3 (IQR, 2–4) days	2 (IQR, 2–4) days
Treatment	Pretreatment with obinutuzumab Tocilizumab: 31 (NR) Steroids: 27 (NR)	Pretreatment with prednisone Other treatments: NR	Tocilizumab: 3 (7.5%) Steroids: 6 (15%) Tocilizumab and steroids: 4 (10%)	Pretreatment with steroids Tocilizumab: 7 (5%)
ICANS	12 (8%)	10 (6.4%)	4 (5%)^∗^	18 (12%)^∗∗^
Grade ≥ 3	3 (3%)	1 (0.6%)	0 (0%)^∗^	4 (3%)^∗∗^
Neutropenia	58 (38%)	34 (21.7%)	26 (28%)	35 (25%)
Grade ≥ 3	41 (37%)	23 (14.6%)	24 (26%)	27 (19%)
Infections	59 (38%)	71 (45.2%)	18 (20%)	71 (49%)
Grade ≥ 3	23 (15%)	23 (14.6%)	13 (14%)	33 (23%)

*Note:* FDA approval was granted for glofitamab and epcoritamab in relapsed/refractory diffuse large B‐cell lymphoma for patients who received ≥ 2 lines of therapy. Mosunetuzumab was approved for relapsed/refractory follicular lymphoma for patients who received ≥ 2 lines of therapy. Odronextamab has not yet obtained FDA approval. Data are no. (%) unless otherwise indicated. ICANS, immune effector cell–associated neurotoxicity syndrome; IQR, interquartile range; IV, intravenous; SQ, subcutaneous.

Abbreviations: CR, complete response; CRS, cytokine release syndrome; FDA, Food and Drug Administration; mDOR, median duration of response; mPFS, median progression‐free survival; NR, not reported; ORR, overall response rate.

^∗^Reported as neurotoxicity.

^∗∗^Reported as ICANS‐like events.

## 3. Infectious Complications

Infectious complications are a well‐established, perpetual toxicity associated with BsAbs, with a significant proportion of recipients developing infections [24–26]. Infectious complications in the trials examining BsAbs have shown an infection risk between 35% and 75% [3–9].

Many infections from the BsAb clinical trials were classified as grade ≤ 2, which may be managed in the outpatient setting. The most frequently reported infections across trials were pulmonary and genitourinary [22]. The incidence of grade ≥ 3 infections ranged between 7% and 45%.

Infectious complications of BsAb have been explored in real‐world studies [27–29]. In patients exposed to teclistimab, for example, authors have reported that 30%–40% of patients have infectious complications. Another study investigating infectious complications in patients with relapse and refractory multiple myeloma reported a similar incidence in patients treated with teclistamab, talquetamab, or elranatamab [30]. Previously reported data suggest that common infectious complications include pulmonary infections, a significant proportion (*n* = 41%) of which are grade ≥ 3 [26]. Jourdes et al. (2024) also reported that most infections in their myeloma BsAb cohort were pulmonary (*n* = 116, 50%). Of all patients who developed an infection (*n* = 234) in that study, 123 (53%) were grade ≥ 3. While high‐grade infections were reported in these real‐world studies, close surveillance in the outpatient setting could allow for timely hospitalization and escalation of care.

The mortality rates attributable to infections can be extrapolated from other related T‐cell therapies in the real‐world setting and may guide management for BsAbs [31–33]. A systematic review reporting nonrelapse mortality after CAR T‐cell therapy, for example, showed that more than half of nonrelapse deaths among CAR‐T recipients were attributed to infections, highlighting the importance of vigilant monitoring and surveillance of infections, frequent follow‐up, and maintaining a low threshold for hospitalization [31].

Of note, infectious complications may vary based on the BsAb target. A study surveying the infectious complications in patients receiving anti‐BCMA agents noted that 45% (27/60) of patients developed an infectious complication by the median follow‐up time, 11.5 months [34]. The median time to infection was 3.3 months, and 48% of first infections occurred within the first 100 days of treatment. Forty‐five percent of the reported infections were documented as pneumonia (22%), respiratory tract infection (13%), or RSV infections (10%). Urinary tract infections were reported as 1.7% of the documented infections. A single‐center study surveying the infectious complications of anti‐CD20 BsAbs noted that 56% (47/84) of patients developed an infectious complication of any sort [35]. Like anti‐BCMA–targeted therapy, CD20‐targeted therapy had a large proportion of patients develop pulmonary infections. Twenty‐three percent of infectious complications were documented as pneumonia, and 21% were documented as upper respiratory tract infection. Unlike anti‐BCMA agents, anti‐CD20 agents had a larger proportion of patients who developed urinary tract infections. Twenty‐one percent of infectious complications were documented as urinary. The time from infection to treatment was 36 days (range 12–84 days). These data suggest that patients on anti‐CD20–targeted therapy may develop infectious complications sooner than those on anti‐BCMA agents. Talquetamab is an anti‐GPRC5D BsAb for which infectious complications have been documented. Infectious complications are reported to be 58.7%, 66.2%, and 72.5% among patients; the rate of infectious complications was dependent on the dosing given to patients [36]. Respiratory infections were a common source of infectious complications in this patient population. Studies comparing the infectious complications between BCMA and GPRC5D suggest that GPRC5D treatment has a lower rate of infections; of the patients who did develop infections, they were also more likely to have a lower‐grade infection when compared to other groups [37]. Studies also suggest that BCMA treatment has a higher rate of infectious complications compared to CD20 treatment [38].

## 4. Current Protocols to Mitigate Toxicity

Recently published consensus recommendations on the management of toxicities associated with CD3×CD20 BsAb therapy provide a working framework for patients with B‐cell non‐Hodgkin lymphoma [23]. Guidelines for the utilization of BsAbs in patients with multiple myeloma have also been published [39]. These guidelines may serve as a framework for other diseases as indications for BsAbs continue to expand.

### 4.1. Management of CRS

CRS is one of the most frequently experienced adverse events in patients treated with BsAbs. CRS is a systemic inflammatory response caused by the activation of T lymphocytes, macrophages, and monocytes secreting cytokines such as interferon‐gamma, interleukin (IL)‐6, and IL‐10. In low‐grade CRS (grade ≤ 2), patients experience fever. However, in higher‐grade CRS (grades ≥ 3), patients experience hemodynamic instability, hypoxia, or end‐organ dysfunction, which require intensive care–level management.

Initial diagnostic workup should include a comprehensive metabolic panel, complete blood count, and lactate dehydrogenase (LDH) levels [23]. Markers of inflammation (baseline cytokine levels, ferritin, C‐reactive protein [CRP], etc.) have an unclear role in predicting CRS/ICANS and should be optional in clinical practice. Baseline cardiac examination and echocardiography should be considered for those with known cardiac comorbidities, given the concerns for poor compensation in the setting of hemodynamic instability associated with high‐grade CRS. Consensus guidelines recommend using the grading system developed by the American Society for Transplantation and Cellular Therapy for determining the severity of CRS [39]. This can be utilized to determine whether or not patients should be admitted for further evaluation.

All approved BsAb products have step‐up dosing to mitigate the risk of CRS. Premedication with dexamethasone or tocilizumab can also be considered to minimize the CRS risk. Predosing with an anti‐CD20 monoclonal antibody may be considered to decrease the CRS risk, according to the BsAb prescription label. Recent studies have explored the role of prophylactic tocilizumab in minimizing the incidence of CRS [40, 41]. This study demonstrated a lower incidence of CRS in patients with multiple myeloma receiving BsAbs without compromising efficacy [40]. Administering tocilizumab prophylactically, therefore, may help facilitate outpatient administration by minimizing this complication.

Other immunomodulatory agents such as antitumor necrosis factor inhibitors like infliximab, adalimumab, and certolizumab could further be utilized. JAK‐STAT inhibitors, such as ruxolitinib and itacitinib, have also demonstrated efficacy in CRS. Anakinra, an IL‐1 receptor antagonist, can also be used in refractory cases.

The patient should remain within proximity of the treating center (preferably within 2 h) or a nearby hospital with critical care facilities, with at least two doses of tocilizumab available on the formulary. Although Grade 1 CRS can be managed in the outpatient setting, grade ≥ 2 CRS should be managed in the hospital setting.

Other strategies, including hydration with intravenous fluids and administration of medications like dexamethasone, acetaminophen, and diphenhydramine, have been utilized in pilot programs administering BsAbs in the outpatient setting with good efficacy in patients with relapsed and treatment‐refractory multiple myeloma [42, 43]. A dexamethasone “pill‐in‐the‐pocket” approach can be given to patients who develop symptoms of mild‐grade CRS. Dexamethasone has demonstrated efficacy in BsAb‐induced CRS [44, 45]. If patients’ symptoms do not improve with this “pill‐in‐the‐pocket,” they can present for further evaluation and hospitalization. A previous study demonstrated that many patients who develop CRS from BsAbs develop low‐grade CRS (93%, *n* = 12/13) and respond to steroids alone [46]. Tocilizumab may also be administered to treat CRS [47]. A study demonstrated that tocilizumab administration in the outpatient setting prevented hospitalization in 15 of 35 patients who developed CRS following CAR‐T therapy [48]. A similar approach may be used in patients receiving other T‐cell–engaging therapies in the outpatient setting.

### 4.2. Management of Neurotoxicity

Neurological adverse events are observed infrequently across BsAb clinical trials and the real‐world data available thus far. Routine neurological testing in asymptomatic patients with an unremarkable neurological examination is not recommended. Patients and caregivers should be educated on potential manifestations of neurological toxicities and encouraged to monitor for any changes that deviate from the baseline. Clinicians can grade neurological changes with an immune effector encephalopathy (ICE) score [49, 50]. This score measures alterations in speech, orientation, handwriting, attention, and receptive aphasia. ICE scores < 7 warrant hospitalization for close surveillance and management [23].

Should neurotoxicity occur, multidisciplinary management should include consultations with the neurologist and intensivist, depending on the severity. For patients with an ICE score of ≥ 7, dexamethasone 10 mg should be given every 12–24 h depending on the severity of symptoms. Some guidelines recommend observation in patients with an ICE score of ≥ 7 [39]. Observation may be performed in the outpatient setting [51]. If symptoms do not improve, patients should be admitted for further workup and management. Clinicians should take a thorough history and physical examination to evaluate alternative etiologies. Further workup can include computed tomography scan of the head, electroencephalogram, magnetic resonance imaging, and/or lumbar puncture. Management should be driven by the clinical presentation. Patients with Grade 1 ICANS can be managed symptomatically with lorazepam or haloperidol. For Grade 2 ICANS, dexamethasone or methylprednisolone can be used. Grade 3 ICANS is managed by administering anti–IL‐6 therapies, such as tocilizumab. For Grade 4 or refractory ICANS, immunosuppressive therapy, such as cyclophosphamide, should be considered.

### 4.3. Management of Infectious Complications and Cytopenia

Cytopenia and infections are also frequent complications experienced in patients receiving BsAbs therapy [22]. Bone marrow suppression is caused by cytokines, which impair hematopoiesis. For patients who develop neutropenia, myeloid growth factor support is recommended for grade ≥ 3 neutropenia [52]. Clinicians should also consider a workup to delineate the etiology of prolonged cytopenia driven by malignancy‐induced marrow infiltration versus sequestration from organomegaly. Therapy interruption may be considered depending upon the severity and duration of cytopenia. With thrombocytopenia, bleeding risk should be considered. Institutional transfusion protocols for the treatment of anemia and thrombocytopenia should be followed. For patients with hypogammaglobulinemia, IVIG has been demonstrated to reduce the risk of infections [53].

In patients who develop infections (any grade), treatment with BsAbs should be interrupted. It is hypothesized that immune cell stimulation in the setting of active infection may heighten the risk and severity of immune toxicity [54]. Guidelines regarding infections should be followed with appropriate therapeutic management and early consultations with infectious disease experts. Finally, prophylaxis against *Pneumocystis jirovecii* and varicella‐zoster virus should be considered to prevent the development of severe infection [55]. For patients with a history of hepatitis infection, specific antiviral therapy is recommended.

Consensus guidelines currently recommend medications for infection prevention [39]. Acyclovir and valacyclovir should be continued throughout treatment to minimize the risk of HSV or VZV infection. Patients are recommended to continue taking this medication for 3 months off treatment or until CD4 counts are greater than 20o/uL. For other viral infections, including CMV and hepatitis B, clinicians should consider entecavir for those who are at risk of reactivation. Patients should take this medication throughout treatment. Likewise, patients are recommended to take Bactrim or Atovaquone throughout treatment to mitigate the risk of developing *Pneumocystis jirovecii* infection. The consensus guidelines recommend antibacterial and antifungal agents for patients who develop neutropenia to minimize the risk of developing bacterial or fungal infections. Lastly, patients who have an IgG concentration < 400 mg/dL should receive IVIG to minimize infectious complication risk. IVIG has been demonstrated to have an increased median infection‐free survival when compared to patients without IVIG supplementation [56]. Patients taking IVIG prophylaxis also had a lower cumulative incidence of ≥  Grade 3 infections when compared to those not taking IVIG prophylaxis (35% vs. 45%). IVIG prophylaxis was found to be clinically beneficial irrespective of IgG levels. Administering IVIG may therefore be beneficial when administering BsAbs in the outpatient setting to minimize infectious complications [56].

## 5. Rationale for Outpatient Management of BsAbs

Given the similarities in immune‐related toxicities between CAR T‐cell therapy and BsAbs, protocols developed for outpatient utilization of CAR T‐cell therapy can serve as a foundation for BsAb outpatient utilization. Bansal et al. [57] evaluated the utility of CAR T‐cell therapy in the outpatient setting and identified variables predictive of early hospitalization (use of bridging therapy, elevated CRP, and LDH). Using available data for outpatient CAR T‐cell administration, a similar framework could be developed to identify high‐risk patients who should undergo inpatient initiation of BsAb therapy, such as patients with a high tumor burden. Furthermore, remote patient monitoring platforms to closely monitor patients for the development of toxicity could be utilized wherever applicable. This has been utilized previously in practice, with patients responding positively to the intervention [58]. Small‐scale studies have been published evaluating the feasibility of administering BsAbs in the outpatient setting [43, 51, 59].

Other groups have produced protocols to demonstrate that T‐cell therapy can be utilized in the outpatient setting safely and effectively. A group of investigators from the University of Oklahoma structured a CAR T‐cell program in an outpatient setting [60]. The authors emphasized developing 8 essential components for a successful outpatient program: (1) construction of a multidisciplinary team, (2) training nursing staff, (3) alerting providers in the community, (4) continuous review of processes and procedures, (5) advancing patient knowledge and facilitating patient support, (6) acquiring physical space for implementation, (7) review of finances, and (8) reevaluation of outcomes and strategy. The investigators monitored patients daily on Days 1–14 and then three times per week on Days 15–28. The providers ensured that the patients stayed within 25 miles of a hospital for the first 2 weeks and then within 100 miles of a hospital for the following 2 weeks. In that single‐center experience, 6 (29%) of 21 outpatients did not require hospitalization within the first 30 days. For the 15 patients who were admitted (71%), the median length of admission was 4 days. A similar study at the University of Pennsylvania reported safe and feasible outpatient administration of CAR T cells, with an admission rate of 9 (31%) of 29 outpatients, with a median time to admission of 5 days after infusion [61]. These single‐center experiences with CAR T cells suggest that many patients can tolerate outpatient initiation. These studies pave the way for further investigation into outpatient implementation of other T‐cell therapies, particularly BsAbs. However, further studies and consensus protocols are warranted.

Investigators at the Mayo Clinic examined the utility of teclistamab for patients with R/R multiple myeloma in the outpatient setting [46]. The authors reported that 24 patients received 223 teclistamab infusions in the outpatient setting. Of these patients, 38% (*n* = 9) had high‐risk cytogenetics. Nearly all doses (220/223; 99%) were given in the outpatient setting, and 204/220 (93%) infusions required no inpatient admission for management. The authors reported that 16 teclistamab infusions resulted in admission, with 4 of 16 infusions requiring readmission for a total of 20 hospitalizations. The most common reason for first‐time admission was fever (*n* = 13/16 [85%]). Overall, 13 patients experienced CRS, with 93% of these events being Grade 1 CRS, managed with corticosteroids alone. Tocilizumab was administered in the outpatient setting for steroid‐refractory CRS [46]. The authors reported a single Grade 4 CRS event, which occurred during dialysis. No other adverse events were reported in the analysis. Although small and single‐centered, the analysis highlighted that teclistamab administration in the outpatient setting was safe and feasible and reduced healthcare resource utilization.

Outpatient bispecific T‐cell engager therapy (blinatumomab) has been administered in the outpatient setting in B‐cell acute lymphoblastic leukemia since its approval in 2016 [62]. Investigators reported that only 22% of patients experienced adverse events that required hospitalization. To ensure adequate safety, monitoring, and timely management, the authors utilized wearable devices, such as the Current Health Wearable Monitoring System. This device provided information regarding continuous oxygen saturation, respiratory rate, and heart rate. The authors also facilitated communication by providing mobile devices to help initiate contact with the healthcare system. These techniques may be utilized for newer BsAbs.

## 6. Potential Framework

**FIGURE 1:**
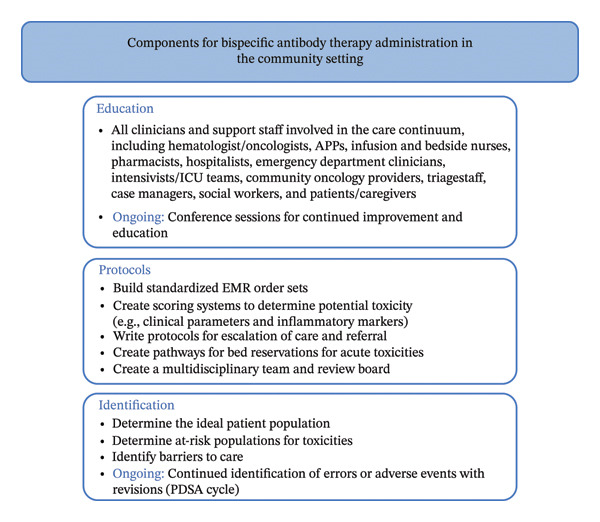
Proposed components for a BsAb therapy infrastructure for community and outpatient settings.

**FIGURE 2:**
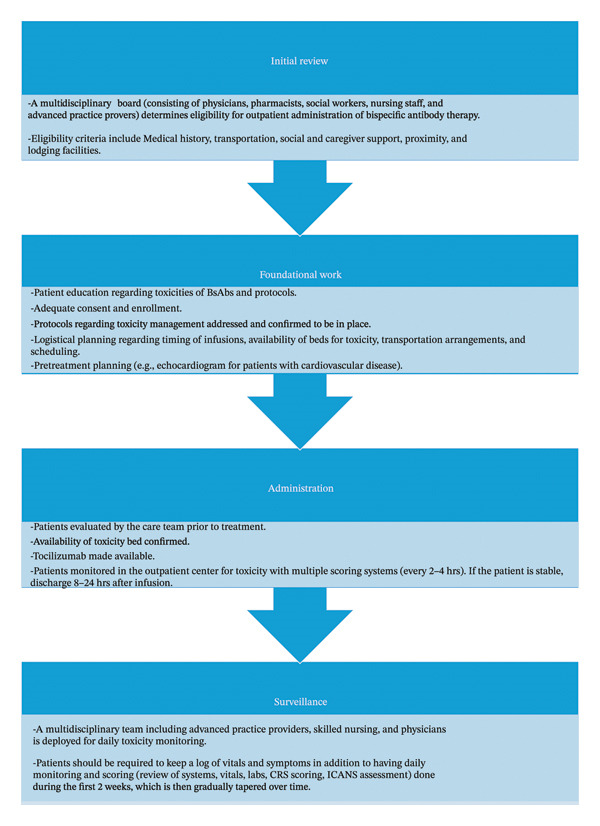
Flowchart for BsAb therapy schema in the community and outpatient settings.

A framework for the safe administration of BsAb therapy in the outpatient and community settings is proposed in Figure 1. Previous literature has also demonstrated some efficacy in implementing these medications in the outpatient setting with manageable toxicities [43]. Figure 2 presents a schema adopted from other groups’ experience with the outpatient CAR‐T therapy administration and serves as a foundation upon which the BsAb outpatient implementation could be structured.

First, focused education for all stakeholders should be prioritized. These stakeholders include physicians, advanced practice providers, pharmacists, nursing staff, patients, and caregivers. Clinical staff should be educated on BsAb therapy and toxicity; institutional protocols should be developed to swiftly escalate care and admit patients for a higher level of care should the need arise [63]. Order sets should be created with a team of electronic medical record developers to standardize workflow, with built‐in templates and protocols to ensure close surveillance. Scheduled toxicity checks may also minimize unexpected complications and allow for timely escalation of care, if needed.

Second, providers should identify the appropriate patient population that would benefit from this framework. Social workers may help identify barriers that would preclude patients from attending frequent appointments. They may also identify resources to assist patients with transportation. Considerations to identify ideal candidates include place of residence, transportation availability, financial status, insurance coverage, access to the internet or phone, and social support systems. Furthermore, clinical characteristics, such as tumor volume and comorbidities, should be strongly considered, as this may increase the risk of complications such as CRS and ICANS [64]. Patient tolerance to prior lines of therapy and previous trials of BsAbs, if applicable, should be utilized to determine candidacy.

Monitoring strategies in the outpatient setting should be tailored to the specific BsAb being administered. Differences in the timing and severity of toxicities, particularly CRS, should guide the duration and intensity of observation following step‐up dosing. For example, agents such as teclistamab are associated with early onset of CRS, often within the first 1–2 days, and may require closer monitoring during initial doses. In addition, certain products have risk evaluation and mitigation strategy (REMS) requirements that may influence monitoring logistics and site of care. As such, outpatient protocols should incorporate product‐specific considerations, including expected timing of adverse events and regulatory requirements, rather than relying on a uniform monitoring approach across all BsAbs.

Finally, a plan–do–study–act cycle should be put into place to continuously advance the model for outpatient BsAbs. Ishikawa (fishbone) diagrams should be conducted to determine potential sources of complications should they arise. With continued improvement, these programs could expand to involve community providers with referrals to academic centers should complications arise.

## 7. Conclusions

Landmark trials have described the distinctive nature and burden of toxicities in patients receiving BsAb therapy. Most toxicities associated with BsAb can be classified as low‐grade and are generally manageable [46]. Furthermore, should significant toxicity occur, protocols are evolving to provide a robust infrastructure for management [23]. Preliminary data evaluating outpatient initiation for other T‐cell therapies, such as CAR‐T therapy, demonstrate feasibility; however, more real‐world studies evaluating this paradigm are needed to further demonstrate safety [46, 57, 58]. By providing outpatient BsAb therapy, physicians and centers can enhance access to care and improve patient satisfaction.

With academic, community, and industrial partnerships, broader deployment of BsAb could be streamlined. Education, provision of in‐services, leveraging protocols and experiences, and development of templates and guidelines will make this transition smoother and allow every patient in need of BsAb to receive timely access to these survival‐prolonging therapies.

## Author Contributions

Muhammad Bilal Abid conceptualized and supervised the project. Amrit S. Gonugunta, Asad Haider, and Muhammad Bilal Abid gathered data. Amrit S. Gonugunta wrote the first draft of the manuscript. Amrit S. Gonugunta, Asad Haider, Virginia Mohlere, Shahrukh Hashmi, Mahmoud Aljurf, and Muhammad Bilal Abid critically revised the manuscript.

## Funding

No funding was obtained for this study.

## Conflicts of Interest

The authors declare no conflicts of interest.

## Data Availability

Data sharing is not applicable to this article as no datasets were generated or analyzed during the current study.
